# USEEIO v2.0, The US Environmentally-Extended Input-Output Model v2.0

**DOI:** 10.1038/s41597-022-01293-7

**Published:** 2022-05-03

**Authors:** Wesley W. Ingwersen, Mo Li, Ben Young, Jorge Vendries, Catherine Birney

**Affiliations:** 1 US Environmental Protection Agency, Office of Research and Development, Washington, USA; 2 General Dynamics Information Technology, Inc, Falls Church, VA 22042 USA; 3 Eastern Research Group, Lexington, MA 02421 USA

**Keywords:** Environmental impact, Industry

## Abstract

USEEIO v2.0 is an environmental-economic model of US goods and services that can be used for life cycle assessment, footprinting, national prioritization, and related applications. This paper describes the development of the model and accompanies the release of a full model dataset as well as various supporting datasets of national environmental totals by US industry. Novel methodological elements since USEEIO v1 models include waste sector disaggregation, final demand vectors for US consumption and production, a domestic form of the model that can be used to separate domestic and foreign impacts, and price adjustment matrices for converting outputs to purchaser price and in various US dollar years. Improvements in modeling national totals of industry and environmental flows are described. The model is validated through reproduction of national totals from input data sources and through analysis of changes from the most recent complete USEEIO model that can be explained based on data updates or method changes. The model datasets can all be reproduced with open source software packages.

## Abstract (summary)

**Table:** 

Measurement(s)	environmental pollution • waste material • air pollution • water pollution
Technology Type(s)	input-output • computational modeling
Factor Type(s)	economic input-output data • environmental release data • resource use data
Sample Characteristic - Location	United States

## Background & Summary

USEEIO v2.0, or referred to solely as v2.0, is the latest edition of the US Environmentally-Extended Input-Output (USEEIO) model for assessing a full suite of potential life cycle impacts of US goods and services. It is the first model version since USEEIO v1.2 capable of calculating potential environmental impacts, resource use and waste generation along with economic impacts, and builds upon the creation of the USEEIO v2 GHG models, which were a series of USEEIO models used to calculate Supply Chain Greenhouse Gas Emission Factors^1^. This paper presents a summary of the complete v2.0 model attributes and model creation with a focus on describing methodological updates since the publication of the original USEEIO methodology.

The first complete and peer-reviewed USEEIO model, v1.0, was released in early 2017 and described in Yang *et al*.^2^ and related datasets^3,4^. This model was based on the 2007 input-output data with 385 commodities and mixed-year environmental data with the latest representing 2013. v1.1 added additional satellite tables and made methodological updates to some existing tables^5,6^. v1.2 added new satellite tables for commercial waste^7,8^.

USEEIO v2.0 described herein is a commodity model with the full breadth of US economic output split into 411 commodity categories. The technical model name for the model described here is USEEIO v2.0.1–411 following the USEEIO versioning scheme as of model finalization^9^, but it is referred to throughout simply as v2.0. The Bureau of Economic Analysis (BEA) 2012 Detail IO tables^10^ define 405 commodity categories. In v2.0, one of the BEA commodities is split into 7 further resolved (more specific) commodities (404 + 7 = 411). A full list of model commodities is presented in the primary data record. v2.0 is a single region model with the 50 states of the United States modeled as a single region. US Territories and Tribal Lands are not included. Domestic and rest of the world (RoW) impacts can be split out in the model calculations.

Representing the most current conditions feasible given the full national economic scope of the model, and yet wanting to retain a high level of resolution to differentiate between US commodities and their life cycle performance, v2.0 is built upon datasets representing the most current years processed from input data sources. The economic data base year for v2.0 is 2012, corresponding to the latest detailed IO tables^10^. These tables are also known as the benchmark tables because they are based on the US Economic Census which is conducted every five years and the tables correspond to the Census year^11^. These tables are typically released 5 or more years after the Census is performed. However, annual tables are also published at a more aggregated level of detail and with less reported data but with only a ~1–2 year time lag to help address the time lag limitations^12^. Many environmental and employment data sources are available to characterize US industries at the needed level of detail for more recent years. These datasets are more current than the IO data, and more closely represent current environmental performance. In v1 models, a base economic year of 2013 – different than the IO year of the economic data – was used. In v1 models, the direct and total requirements were determined from analysis to be adequate in representation of 2013 conditions (see SI1 from Yang *et al*.)^2^, therefore all environmental data was adjusted to be in 2013 US Dollars (USD). For v2.0, the USD year of the model is the same as the IO data, 2012 USD.

v2 models represent a second generation of USEEIO models built using an improved technical infrastructure^9^. A set of ~40 v2 models with only greenhouse gas (GHG) satellite tables were constructed using IO and environmental data for varying years (2010–2016), number of sectors (~400, ~70, ~15) and model formulation types (commodity-based and industry-based) to generate supply chain GHG emission factors^1^. However, these models did not incorporate additional sector disaggregation, did not include domestic model variants, lacked other environmental matrices and associated indicators described for v2.0 herein, and the full set of matrices for these models were not published. These novel elements as well as model fundamentals are described in this paper.

## Methods

This section describes data sources, algorithms for model construction, novel methodologies and software procedures used in the construction of USEEIO v2.0.

### Summary of economic, environmental and indicator method inputs

The 2012 BEA Detail Make and Use Tables Before Redefinitions in Producer’s Price are used as the underlying IO tables. Producer price is the price of a commodity reflecting an industry’s cost to produce it including commodity taxes^11^. Other economic data sources used include the Gross Industry Output data and the associated Gross Output Chain-Type Price Index data for years 2002–2017, the 2012 Margins data that contain the value added per commodity between point of manufacture and point of sale that make up the difference between producer’s and purchaser’s price, and the 2012 Import Matrix (Table 1).

**Table 1:** Economic Data^76^.

Name	Creator	Sources	DataYears
Make and Use, Detail, Before Redefinitions, Producer Price	BEA	Bureau of Economic Analysis Industry Input-Output Accounts	2012
Gross Output by Industry	BEA	Gross Output By Industry	2002–2017
Gross Output Chain Price Index	BEA	Gross Output By Industry	2002–2017
Margins	BEA	Bureau of Economic Analysis Industry Underlying Estimates	2012
Import Matrix	BEA	Bureau of Economic Analysis Industry Input-Output Accounts	2012

National totals of flows (physical movements of specific resources, emissions or employment) by industries are used as the sources of environmental and employment data. Coverage of these data used in v2.0 is equivalent to that from v1.2 as seen in Table 2. These include all the types of resource use and environmental releases/losses from v1.1^5^ plus the three additional waste generation datasets created for v1.2^7,8^. The data for water withdrawals, criteria and hazardous air emissions, point source industrial releases to ground, point source releases to water, greenhouse gases, land use, employment, and value added have been updated in v2.0 and incorporate methodological improvements. The breakdown of these data into the records given in Table 2 is not identical to that given in Table 1 of the original USEEIO description^2^, but all these data are aggregated during model construction (see Model Construction section), and therefore the breakdown just describes the form of these data as they are originally processed and imported. Changes in selection of data sources and methodologies for compiling these into a standard format are described below. New procedures for preparing and integrating these datasets into the model are described in the Procedure for Model Building section.

**Table 2:** Environmental and Employment Data Inputs.

Name	Code	Creator	Sources	DataYears
Water withdrawals	WAT	USGS	Water Use in the US^45^	2015
Criteria and Hazardous Air Emissions	CHAIR	USEPA	National Emissions Inventory^28^; Toxics Release Inventory^29^	2017; 2017
Point source industrial releases to ground	GRDREL	USEPA	Toxics Release Inventory^29^	2017
Point source releases to water	WATREL	USEPA	Toxics Release Inventory^29^; Discharge Monitoring Report^33^	2017; 2017
Greenhouse Gases	GHG	USEPA	GHG Inventory^69^	2016
Land use	LAND	BLM; EIA; EIA; USDA	Public Land Statistics^77^; Commercial Building Energy Consumption Survey^38^; Manufacturing Energy Consumption Survey^30^; Major Uses of Land in the United States^36^	2012; 2012; 2014; 2012
Mineral extraction	MINE	USGS	Mineral Commodity Summary^78^	2014
Energy extraction	ENERGY	EIA	Monthly Energy Review^79^; Form EIA-923 Detailed^80^	2014
Nitrogen and Phosphorus Releases from Agriculture	NPAG	USDA	Chemical Use Survey^81^	2014; 2010; 2011; 2013; 2012; 2014
Pesticide releases	PEST	USDA	Chemical Use Survey^81^	2014; 2010; 2011; 2013; 2012; 2014
Commercial non-hazardous waste excluding construction activities	CNHW	CalRecycle	2014 Generator-Based Characterization of Commercial Sector^82^	2014
Commercial non-hazardous waste from construction activities	CNHWC	USEPA	Advancing Sustainable Materials Management: 2014 Fact Sheet^83^	2014
Commercial RCRA-defined hazardous waste	CRHW	USEPA	National Biennial RCRA Hazardous Waste Report^56^	2017
Employment	EMP	BLS	Quarterly Census of Employment and Wages^58^	2017
Value Added	VADD	BEA	Detail Use Before Redefinitions^76^	2012

The model indicators quantitatively relate the environmental and economic flow data to an aggregate impact through the use of characterization factors. These include values such as the carbon dioxide equivalencies of the flows that are greenhouse gases. The same indicators used in v1.1^13^ along with the three indicators (CNHW, CNHWC, CRHW) for waste generation^8^ are used in v2.0 (Table 3).

**Table 3:** Indicator Data Inputs.

Name	Code	Creator	Sources
Greenhouse Gases	GHG	USEPA	TRACI 2.1^84^
Acidification Potential	ACID	USEPA	TRACI 2.1
Eutrophication Potential	EUTR	USEPA	TRACI 2.1
Freshwater Ecotoxicity Potential	ETOX	USEPA	TRACI 2.1
Human Health - Cancer	HCAN	USEPA	TRACI 2.1
Human Health - Noncancer	HNCN	USEPA	TRACI 2.1
Human Health Toxicity	HTOX	NA	Aggregation of HNCN and HCAN
Human Health - Respiratory Effects	HRSP	USEPA	TRACI 2.1
Ozone Depletion	OZON	USEPA	TRACI 2.1
Smog Formation Potential	SMOG	USEPA	TRACI 2.1
Freshwater withdrawals	WATR	USEPA	FEDEFL Inventory Methods v1.0.0^85^
Land use	LAND	USEPA	FEDEFL Inventory Methods v1.0.0
Hazardous Air Pollutants	HAPS	USEPA	FEDEFL Inventory Methods v1.0.0
Pesticides	PEST	USEPA	FEDEFL Inventory Methods v1.0.0
Nonrenewable Energy Use	NNRG	USEPA	FEDEFL Inventory Methods v1.0.0
Renewable Energy Use	RNRG	USEPA	FEDEFL Inventory Methods v1.0.0
Energy Use	ENRG	USEPA	FEDEFL Inventory Methods v1.0.0
Minerals and Metals Use	MNRL	USEPA	FEDEFL Inventory Methods v1.0.0
Value Added	VADD	USEPA	USEEIOv1.1 - Elementary Flows and Life Cycle Impact Assessment (LCIA) Characterization Factors^13^
Jobs Supported	JOBS	USEPA	USEEIOv1.1 - Elementary Flows and Life Cycle Impact Assessment (LCIA) Characterization Factors
Commercial RCRA Hazardous Waste	CRHW	USEPA	Commercial Waste National Totals by NAICS and US Satellite Tables for USEEIO^7^
Commercial Municipal Solid Waste	CMSW	USEPA	Commercial Waste National Totals by NAICS and US Satellite Tables for USEEIO
Commercial Construction and Demolition Debris	CCDD	USEPA	Commercial Waste National Totals by NAICS and US Satellite Tables for USEEIO

New for v2.0 is the use of a standard flow list for representing elementary flows, or raw materials from or returning to the technosphere. The Federal LCA Commons Elementary Flow List (FEDEFL) v1.0.7 is used to represent the substance, environmental compartment or origin or release, and the unit in a common format with Federal LCA data^14,15^. Additionally, to support the use of the FEDEFL for the new environmental data, flows used in the indicators also were updated to correspond to the FEDEFL. This flow update afforded the opportunity to use more standard life cycle impact assessment (LCIA) characterization factors to populate these indicators, which were integrated with the new procedure. More information about this update is provided in the Procedure for Model Building section.

Three standard national level demand vectors were created for use with the model to calculate potential impacts of US consumption, production and consumption from households. Each demand vector was derived from the BEA Detail 2012 Use table. Derivation of these demand vectors is described in depth in the Final Demand section, since these have not been previously described in USEEIO documentation.

### Model construction

Matrix algebra is used to represent the steps of creating the USEEIO model, using conventions for variable names commonly used in a mix of standard references for IO analysis^16^ and LCA^17^, and the existing USEEIO model documentation. Capital letters indicate matrices and lower case letters indicate vectors. A “^” symbol over a variable represents the diagonalization of a vector as a matrix. An exponent of “−1” represents an inverse. A “′” symbol represents the transposed (rows and columns switched) form of a matrix or vector. The operator ‘⚬’ is used for elementwise multiplication in contrast with no symbol between adjacent matrices or vectors which denotes a matrix multiplication. All nomenclature used is defined in the Table 4.

**Table 4:** Variables.

Variable	Contents	Shape
A	direct requirements matrix	sector x sector
B	satellite table/stressor matrix	flow x sector
C	characterization factor matrix	indicator x flow
c	used as subscript for commodity	
D	direct impact matrix	indicator x sector
d	used as subscript for domestic	
E	environmental totals matrix	flow x sector
e	subscript for exports	
f	subscript for final perspective	
G	direct + indirect total flow matrix, aka Life cycle inventory result	flow x sector
g	subscript for government	
H	direct + indirect total impact matrix, aka Life cycle impact assessment result	indicator x sector
h	direct + indirect total impact vector, aka Life cycle impact assessment result	indicator
h	subscript used for households	
I	Identity matrix	varies
i	vector of 1 s	varies
i	subscript used for industries	
L	Leontief matrix	sector x sector
M	direct + indirect flows matrix	flow x sector
m	subscript for imports	
N	direct + indirect impact matrix	indicator x sector
n	subscript for indicator	
P	commmodity by margin type coefficient matrix	sector x year
pro	subscript for producer price	
pur	subscript for purchaser price	
q	commodity output	commodities
r	subscript used for direct perspective	
RoW	subscript for Rest of World region	
s	scaling vector	sector
t	subscript used for transportation	
U	Use table	commodity x industry
V	Make Table	industry x commodity
v	subscript for investment	
w	vector for a weighted impact score calculated for rankings	
w	subscript used for wholesale	
x	industry output	industries
Y	final demand matrix	sectors x final demand component
δ	subscript for change in inventories	
Φ	price type adjustment ratio matrix	sector x year
ϕ	price ratio vector	sectors
Rho (P)	price year adjustment matrix	sector x year
ρ	price adjustment factor	sector
^	symbol that indicates diagonalized form (matrix form) of a vector	
′	symbol that indicates the transposition of a matrix or vector	

#### Model matrices

The core of an IO model is a direct requirements matrix, *A*, representing the dollar inputs from other sectors per dollar output. *A* is created from the normalized forms of the Model Make, *V*, and Use, *U*, tables. The Make table is an industry x commodity matrix with USD annual output of commodity *i* produced per industry *j* in USD; the Use table is commodity x industry matrix with amount of commodity *i*, in USD, used to by industry *j* to provide a good or service. The Make table is normalized by the commodity output vector, *q* to create what is also known as the market shares matrix. The Use table is normalized by the industry output vector, *x*, to result in a commodity x industry direct requirements matrix. The results of those normalizations are multiplied with an orientation to create a commodity x commodity direct requirements matrix, or *A*, using Eq. 1. This method for creating the *A* matrix is based on the *industry- technology* assumption, wherein the manufacture of the primary and any secondary commodities by an industry uses the same production requirements, and the commodity requirements are based therefore on the mix of industries that produce that commodity, weighted by their relative share of total commodity output^16^.

1 $$A=U{\widehat{x}}^{-1}V{\widehat{q}}^{-1}$$

*L*, the Leontief inverse, or the total requirements matrix, is obtained from *A*, using Eq. 2. *L* is in commodity x commodity form and represents the total inputs of commodities (rows) used to make a commodity (columns).

2 $$L={(I-A)}^{-1}$$

To relate the environmental and employment data to the IO data, Eqs. 3–6 are used to create the satellite matrix of environmental flow coefficients, *B*, in a physical unit per dollar output for a commodity in the model that can be used with the economic data for industries from another year. Household emissions or other final use emissions are not included in *B*.

3 $$B={B}_{I}V{\widehat{q}}^{-1}$$

In Eq. 3, *B* is in flow x commodity form after transforming *B*_*I*_ into this form with the market shares matrix transformation. The original relation between the environmental data in the form of national totals by industry, *E*, and the model economic data uses the model industry output, as described in Eq. 4.

4 $${B}_{I,y}={E}_{I,z}{\widehat{x}}_{z,y}^{-1}$$

In Eq. 4, *E*_*I*_ is a emission x industry matrix of national totals of each flow by industry sector in year *y*, and *x*_*z,y*_ is a vector of gross output by industry in year *z*, given in year *y* dollars. The industries in the *E* columns match the industries in *x*.

For *x*_*z*_ to be in year *y* USD, the year of the IO data, *x*, must first be price adjusted using Eq. 5, where *x*_*i,z*_ is the year industry output for industry *i* in the currency year, *z*, corresponding to the year of the national flow totals.

5 $${x}_{i,y}={x}_{i,a}\ast {\rho }_{i,z- > y}$$

*ρ* is a price adjustment factor for industry *i* from currency year *z* to *y* USD. *ρ* for industry *i* is prepared using Eq. 6, where *ρ* from currency year *z* to *y* is the ratio of the industry gross output chain price index, *pi*, for year *y* to that of year *z*.

6 $${\rho }_{i,z- > y}=\frac{p{i}_{i,y}}{p{i}_{i,z}}$$

Equations 5, 6 are used for each industry and unique year of environmental data in the given model. The resulting coefficients from these calculations can be interpreted as a measure of the environmental intensity of a sector in the year the environmental data are reported, but given in terms of the IO year dollar value.

The model indicators are put in the form of an indicator x flow matrix, *C*, where the values are the quantitative relation of the flows to the indicator value, also known as characterization factors in the life cycle impact assessment literature.

A series of coefficient matrices are provided that are products of combining more than one of the economic, physical flow, and indicator components. The direct impacts of a sector in a given indicator unit per model dollar year, can be calculated with Eq. 7. *D* is an indicator x sector matrix and contains in each column *i* the direct impact result per 1 USD output of sector *j*.

7 $$D=CB$$

With the flow coefficient matrix *B* and the total requirements matrix *L*, the matrix *M* which contains the direct and indirect flow coefficients can be calculated with Eq. 8. The matrix *M* is a flow x sector matrix and contains in each row *i* the direct plus indirect flows per 1 USD output of the sector in column *j*.

8 $$M=BL$$

With the direct impacts *D* and the total requirements *L*, the matrix *N* which contains the direct plus indirect impact coefficients can be calculated via Eq. 9. *N* combines each economic, flow and indicator component. *N* is an indicator x sector matrix and contains in each row *i* the direct and indirect impact result per 1 USD output of sector *j*.

9 $$N=DL$$

Two matrices are provided that enable price adjustments in the model coefficient matrices (B, D, M, or N).

Model coefficient matrices can be converted to reflect different currency years. *P* is a commodity x year currency year adjustment matrix. The values in *P* are commodity-specific deflation ratios where a value of *P*_*c,y*_ is a ratio of model USD:year *y* USD for commodity *c*. Values in *P* are prepared with Eq. 6 above, but using the commodity gross output chain price index in place of the industry gross output chain price index.

Model coefficient matrices may be obtained in purchaser’s price through adjustment of values from producer’s price. Purchaser price reflects the producer’s price plus sale and transportation margins^11^. *Φ* is a commodity x year price type adjustment matrix prepared using Eq. 10. As the matrix values are in producer price USD, the values in *Φ* are commodity-specific producer:purchaser price ratios where a value of *Φ*_*c,y*_ is a ratio of year *y* USD producer price:year *y* USD purchaser price for commodity *c*.

10 $${\varPhi }_{c},y=\frac{{q}_{PRO,c,y}}{{q}_{PUR,c,y}}$$

Margins tables provide values for transportation, *t*, wholesale, *w*, and retail, *r*, specific to each commodity consumed by industries, households or investors. These same year margins data are used for all years, however they are price-adjusted first to calculate a total year specific margin using Eq. 11.

11 $${q}_{PUR,c,y}={q}_{c}{P}_{c,y}+{t}_{c,y}{P}_{t,y}+{w}_{c,y}{P}_{w,y}+{r}_{c,y}{P}_{r,y}$$

where *P*_*m,y*_ for a margin type (*t*, *w* or *r*) is calculated in Eq. 12, which is the weighted average of price adjustments in commodities that make up *m* (e.g. Truck transportation, Water transportation, Rail transportation are commodities in set *m* for transportation).

12 $${P}_{m,y}=\frac{{\sum }_{c\in m}{q}_{c,y}{P}_{c,y}}{{\sum }_{c\in m}{q}_{c,y}}$$

#### Final demand

The final demand vectors represent purchases of goods and services by final consumers, including by households, investors and governments. The BEA Use table reports the data for final US demand by these consumers, grouping them at varying levels of resolution depending on the level of resolution of the Use table (i.e., sector, summary or detail). The Use table also includes imports, exports, and change in inventories of final commodities as a part of final demand.

For v2.0, we derive two primary final demand vectors, a production vector and a consumption vector. We define consumption as final use within the US of all goods and services that are both produced and sold within the US or imported. We define production as final use, either within the US or abroad, of all goods and services that are produced in the US.

In general, the final demand in the BEA Use table can be grouped into the following categories:

*y*_*h*_ = household consumption

*y*_*v*_ = investment

*y*_*g*_ = federal, state and local government consumption

*y*_*m*_ = imports

*y*_*e*_ = exports

*y*_*δ*_ = change in inventories

Depending on the table resolution, these categories are further divided into one or more subcategories. Table 5 provides a list of all final demand columns included in the BEA Detail Use table and their respective association with the final demand variable.

**Table 5:** Groupings of final demand columns in Detailed Use Table.

Variable	Name of column in Use Table
y_h_	Personal consumption expenditures
y_v_	Residential private fixed investment
	Nonresidential private fixed investment in structures
	Nonresidential private fixed investment in equipment
	Nonresidential private fixed investment in intellectual property products
y_g_	Federal national defense: Consumption expenditures
	Federal national defense: Gross investment in equipment
	Federal national defense: Gross investment in intellectual property products
	Nondefense: Consumption expenditures
	Federal national nondefense: Gross investment in structures
	Federal national nondefense: Gross investment in equipment
	Federal national nondefense: Gross investment in intellectual property products
	State and local: Consumption expenditures
	State and local: Gross investment in structures
	State and local: Gross investment in equipment
	State and local: Gross investment in intellectual property products
y_m_	Imports of goods and services
y_e_	Exports of goods and services
y_δ_	Change in inventories

Notable in the BEA data is that imports in *y*_*m*_ are represented with negative values. The totals for household, investment, and government consumers in the Use table include consumption of direct imports as positive values. Also, change in inventories, *y*_*δ*_, is positive for commodities produced but not sold, and negative for commodities consumed from a previous years’ production.

The consumption vector is defined in Eq. 13.

13 $${y}_{c}={y}_{h}+{y}_{v}+{y}_{g}$$

The production vector is defined in Eq. 14.

14 $${y}_{p}={y}_{c}+{y}_{e}+{y}_{m}+{y}_{\delta }$$

The production vector adds to the consumption vector the net trade balance as well as inventory/stock changes.

In the production vector, the direct imports both used by final consumers and industries are removed. When imports are greater than final consumption and exports for a given commodity, the demand value will be negative. However when this demand vector is applied to the model, output of these commodities is positive due to industry consumption, reflecting the commodity output totals.

### Model result calculations

Total flows or impacts associated with a given amount of final demand are calculated using two perspectives that produce the same overall flow or impact totals but associate the totals with different sectors. The *direct* perspective calculation associates the totals with the sectors that produce the given flows (e.g. direct emissions, waste generation, or resource use). These calculations of total flows or impacts associated with a given final demand are equivalent to life cycle inventory (LCI), denoted as *G*, and life cycle impact assessment (LCIA) result, denoted as *H*, in the field of life cycle assessment^17^. The *direct* perspective LCI, denoted as *G*_*r*_, is calculated with Eq. 15.

15 $${G}_{r}=B\widehat{s}$$

where *G*_*r*_ is the *direct* + *indirect* flows x sector matrix and *s* is a scaling vector. *s* is the product of *L* and the given final demand vector, *y*, as shown in Eq. 16.

16 $$s=Ly$$

A similar approach is used to calculate the *direct* + *indirect* impacts x sector with the direct perspective as *H*_*r*_ but it uses includes the *D* direct impact matrix to characterize those flows as shown in Eq. 17.

17 $${H}_{r}=D\widehat{s}$$

The *final* perspective associates the totals with the final consumption sectors that drove that impact. The *direct* + *indirect* flows x sector matrix with the final perspective, *G*_*f*_ is calculated with Eq. 18.

18 $${G}_{f}=M\widehat{y}$$

The *direct* + *indirect* impacts are calculated like in Eq. 18, but use the *N* matrix in place of *M*, as shown in Eq. 19.

19 $${H}_{f}=N\widehat{y}$$

Assessing the flows or processes (i.e. sectors in an EEIO model) that drive a particular indicator value is a conventional analytical practice in life cycle assessment^18^. The relative contribution, *rc* of a flow, *f*, to an impact intensity coefficient from *N* for a given indicator, *n*, can be calculated using Eq. 20.

20 $$r{c}_{f},n=\frac{{m}_{f}\circ {c}_{n}^{{\prime} }}{\sum \left({m}_{f}\circ {c}_{n}^{{\prime} }\right)}$$

where *m*_*f*_ is the column representing the flow of interest from the *M* matrix, and *c*_*n*′_ is the transposed row representing the indicator of interest from the *C* matrix.

The relative contribution, *rc* of a commodity, *c*, to an impact intensity coefficient from *N* for a given indicator, *n*, can be calculated using Eq. 21.

21 $$r{c}_{c},n=\frac{{l}_{c}\circ {d}_{n}^{{\prime} }}{\sum ({l}_{c}\circ {d}_{n}^{{\prime} })}$$

where *l*_*c*_ is the column representing the commodity of interest from the *L* matrix, and *d*_*n*′_ is the transposed row representing the indicator of interest from the *D* matrix.

### Domestic matrices

In order to split impacts between US and Rest of World (RoW), the requirements from production need to be split between domestic inputs and foreign inputs. This can be performed by subtracting the import matrix, *U*_*m*_ from the Use matrix to estimate a domestic Use table, *U*_*d*_, as in Eq. 22.

22 $${U}_{d}=U-{U}_{m}$$

Then, the imports, *y*_*i*_, are subtracted from the final demand in the original Use table, to get domestic final demand, *y*_*d*_, as in Eq. 23.

23 $${y}_{d}=Y-{y}_{i}$$

The *A* matrix with just domestic direct requirements, *A*_*d*_, can be created using a similar derivation used for *A* but using the domestic form of the Use matrix. Note that normalized Make table transactions are unchanged. Equation 24 is given below for the commodity form of the model.

24 $${A}_{d}={U}_{d}{\widehat{x}}^{-1}\ast V{\widehat{q}}^{-1}$$

Then one can continue to derive the equivalent of *L* for domestic use, *L*_*d*_ from *A*_*d*_, using Eq. 25.

25 $${L}_{d}={(I-{A}_{d})}^{-1}$$

#### Splitting impacts between US and RoW

For calculating any domestic result (see sec. 3.3), the *L*_*d*_ and a demand vector derived from *Y*_*d*_ are used. For national USEEIO models, results calculated with these variables represent US region results.

To derive Rest of World region results, the difference between any full result calculation and the domestic calculation can be taken, as in Eq. 26.

26 $${H}_{RoW}=H-{H}_{d}$$

where *H*_*RoW*_ is the contribution from Rest of World, and *H*_*d*_ is the contribution from the US.

### Scrap handling

In v1, the *Scrap* commodity was removed from the model following a methodology presented by BEA for deriving a total requirements matrix^11^. In v2.0, *Scrap* is left in the model to simplify the accounting procedures, but we do not recommend use of multipliers generated from *Scrap* because of the lack of a clear material or functional characterization of this commodity.

### Disaggregation of the waste and remediation services sector

The 2012 BEA IO tables include the sector *Waste management and remediation services* (562000), both as an industry and commodity. This sector provides a wide range of services from non-hazardous waste landfilling and recycling to contaminated site remediation. This level of aggregation prevents targeted analysis of various waste handling activities, such as material recovery (recycling). To provide a greater resolution, a technique commonly applied to IO tables is disaggregation^19^, which splits a single sector into multiple sectors within the IO tables.

The BEA IO sector codes are based on the North American Industrial Classification System (NAICS). The three zeroes at the end of the BEA code for *Waste management and remediation services* indicate that it is at the 3-digit NAICS level. The approach used to disaggregate this sector provides 6-digit NAICS granularity, which is the most detailed NAICS designation given in the official classification. For v2.0, *Waste management and remediation services* is disaggregated into the seven sectors shown in Table 5.

A main assumption in the disaggregation of waste management sectors is that the receivers of waste flows are being paid for waste treatment. In other words, the monetary and physical flows occur in the same direction: makers of the waste treatment commodity receive both the physical waste to be disposed and the money for the disposal service. This is a necessary first order approximation since we found no publicly available data to confirm this for the disaggregated waste sectors.

The disaggregation process is carried out by disaggregating distinct sections of the Use and Make tables. For each of the tables, these sections are the table rows, columns, and intersections. These sections are disaggregated sequentially, and all the disaggregated components of the tables are combined at the end of the process.

#### Data sources for waste sector disaggregation

The US Economic Census (EC), published by the US Census Bureau, provides economic data for all sectors of the US economy and is used to estimate industry consumption of the disaggregated waste management commodities (i.e., Use table rows)^20^. This dataset tracks the monetary receipts by the different waste management subsectors that broadly correspond to the disaggregated sectors being introduced to the v2.0 model.

The Service Annual Survey (SAS) from the US Census Bureau provides estimates of revenue and expenditure data for most traditional service industries^21^, and is used in the disaggregation of the waste management industries (i.e., Use table columns).

The Resource Conservation and Recovery Act Information System (RCRAInfo) database contains information on the flows of hazardous waste^22^, and is mainly used in the disaggregation of the Use table intersection. The information is tracked by how facilities manage hazardous waste – as generators, transporters, or treatment, storage, and disposal facilities (TSDF). For waste management disaggregation, a subset of the RCRAInfo database that contains waste flows from shipping facilities to receiving/storage facilities (arranged by NAICS sector codes) was used. This subset describes the waste flows from the receiving facilities’ point of view, ideal for tracking the information regarding how the waste is managed. This data is more complete than the flows reported by hazardous waste shippers, and so is preferred for the waste disaggregation.

#### Disaggregation of the use table

##### Disaggregation of the use table intersection

The Use table intersection represents the consumption of the *Waste management and remediation services* commodity by the *Waste management and remediation services* industry itself. The major challenge with this section is establishing accurate IO transactions between the disaggregated sectors by using both monetary and material flow data. These data are difficult to obtain given the scarcity in publicly available waste management pricing data, the level of aggregation of the waste management sectors, and the differences in prices and materials used by each waste management activity. The IO transactions for the Use table intersection are assigned based on material flows between the disaggregated sectors, using RCRAInfo data as the main data source. RCRAInfo data are available by 5- and 6-digit NAICS codes, which map to the disaggregated sectors as shown in Table 6. The 5-digit NAICS in the RCRAInfo codes do not count flows present in the 6-digit codes. For example, the total flows counted by the 5-digit *Waste collection* (56211) sector do not equal the flows of the 6-digit *Waste collection* flow subsectors (562611, 562112, 562119). Thus, the flows from the 5-digit sectors must be allocated to the USEEIO sectors. Where a 5-digit NAICS contains only a single 6-digit child NAICS (e.g., 56291), flows are automatically assigned to that sector. Where multiple 6-digit NAICS are present, the following assumptions are used:

- 56211: Most of the flows are to the *Hazardous waste collection* sector (more detail on this below). Thus, whatever is not explicitly allocated to 562112, *Hazardous waste collection*, is assumed to go to *Solid waste collection*, 562111.
- 56221: Most of the flows are to *Hazardous waste disposal* sector. Thus whatever is not explicitly allocated to 562211, *Hazardous waste disposal*, is assumed to go to *Solid waste landfilling*, 562112.
- 56299: All 6-digit NAICS codes under the 5 digit 56299 code are assigned to 562OTH in the USEEIO classification

**Table 6:** Sectors replacing *Waste management and remediation services* (562000) in the disaggregated version of USEEIO.

Description	NAICS Codes	USEEIO Codes
Solid waste collection	562111	562111
Hazardous waste collection treatment and disposal	562112, 562211	562HAZ
Solid waste landfilling	562212	562212
Solid waste combustors and incinerators	562213	562213
Remediation services	562910	562910
Material separation/recovery facilities	562920	562920
Other waste collection and treatment services	562119, 562219, 56299	562OTH

Accordingly, the waste shipped from and to waste management sectors are used as the basis for the mapping of the waste management intersection in the Use table. Using these assumptions, the waste flows between the disaggregated waste management sectors are divided by the total waste shipped between 562 sectors (as indicated in the RCRAInfo data) to obtain a percent allocation value. These values are included the *WasteDisaggaregation_Use* sheet in the primary data record in the *Use table intersection* rows.

##### Disaggregation of the use table columns

The Use table columns represent the inputs that the IO industries need to produce their output. For the disaggregated waste management sectors, this represents the materials and services that these industries consume in the process of collecting, managing, and remediating wastes. Out of the total inputs to the waste management industry, 51% is due to value added sectors, with employee compensation being 29%, suggesting that waste management is a labor-intensive sector. The allocation of the value added sectors is handled separately (and described below). If value added inputs are excluded, the biggest input is the *Waste management and remediation services* sector itself, representing 19% of all intermediate inputs. This suggests that trade flows between the disaggregated sectors is an important component of the aggregate sector’s input, and its disaggregation was previously discussed. After waste management itself, the next 31 input sectors (out of 177) comprise 70% of intermediate inputs.

The SAS data provides the total expenses of more detailed sectors within *Waste management and remediation services*. This information is used to perform a default allocation of the expenses of the disaggregated waste management sectors along the Use table columns, except for the waste management sectors intersection and the value-added sectors. In a default allocation, all columns are disaggregated according to the default percentage values for each of the disaggregated sectors. That is, for each row in the waste management columns, the original value is multiplied by these default percentages and assigned to the corresponding disaggregated column along that row. The columns can later be modified with assumptions for individual commodity expenditures by the disaggregated waste management sectors as additional data is found. These values can be found in the *WasteDisaggaregation_Use* sheet of the primary data record (in the rows labeled *Use column sum*).

##### Disaggregation of the use table rows

The Use table rows represent the use of commodities by the industries in the IO table. For the disaggregated waste management sectors, these rows represent the use of the disaggregated waste management service commodities by industries. The values for the disaggregated waste commodities are allocated based on allocation factors derived from the Economic Census customer classes, as described below.

There are 398 industries and three final demand sectors that consume the *Waste management and remediation services* commodity. Waste commodity consumption is concentrated within a few sectors, with 55% of the commodity consumed by the top five sectors. The largest consumers are residential users (F01000, *Personal consumption expenditures* final demand) at 24% of total consumption; state and local governments (GSLGO, *State and local government and other services*) at 13% of total consumption; and *Other real estates* (531ORE) and the *Waste management and remediation services* sector itself, both at 8% of total consumption.

The Economic Census data provides monetary receipt values by detailed NAICS codes and customer class. Each NAICS sector was matched by customer type, by aligning the NAICS definition of each sector to the customer type it most closely resembled:

- The four sectors pertaining to the federal government (S00500, S006000, S00101, and S00102) were assigned to the ‘Federal government’ customer class;
- The six sectors pertaining to local governments (GSLGE, GSLGH, GSLGO, S00201, S00202, and S00203) were assigned to the ‘State and local governments’ customer class;
- Three sectors (813100, 813A00, and 813B00) were assigned to the ‘Not-for-profit organizations’ customer class;
- The F00100 sector was assigned to the ‘Household consumers and individuals’ customer class;
- All other industries in the USEEIO model are mapped to the ‘Business firms and farms’ customer class.

To obtain an allocation percentage for the industries that consume *Waste management and remediation services* commodity (i.e. the Use table rows), the value of the receipts received by each disaggregated waste management sector by each Economic Census customer class was divided by total value spent by that customer class on *Waste management and remediation services*. For example, the *Solid waste collection* subsector received approximately $21 billion in receipts from the ‘Business firms and farms’ customer class. This class in turn spent a total of almost $48 billion in services from the *Waste management and remediation services* sector. Accordingly, the allocation factor to the disaggregated *Solid waste collection* rows is approximately 44% (21/48) of the total *Waste management and remediation services* commodity consumption for all USEEIO industries mapped to the ‘Business firms and farms’ customer class. The allocation percentages for the consumption of the disaggregated waste commodities by the IO industries are included in the *WasteDisaggaregation_Use* sheet of the primary data record, in the *Use Row Sum, Commodity Output* rows for the ‘Business firms and farms’ customer class and in the *Commodity disaggregation* rows for the other customer classes.

It is worth noting that the Imports and Exports IO sectors (F00500 and F00400) are not classified as any customer class in the Economic Census, but are allocated as ‘Business firms and farms’ for disaggregation purposes. However, the overall effects of any allocation scheme to the imports and exports sectors is fairly small, as they account for a small portion of total commodity use.

##### Disaggregation of value added

The value added sectors are the wages, taxes, and gross operating surplus for the industries present in the USEEIO model. While not part of the interindustry transactions, these sectors are somewhat analogous to commodities, and are represented as rows for each industry in the Use table. The large amount of waste flows shipped to and from the *Hazardous waste treatment and disposal* sector makes this sector dominate the allocation of the use table intersection. 58% of the value of the original waste sector is allocated to the intersection of *Hazardous waste treatment and disposal* with itself, while the *Hazardous waste treatment and disposal* column contains 89% of the total value of the intersection disaggregation. This disproportionate share of the original value of the waste remediation industry, combined with the industry allocations already used for the Use table columns, can result in an imbalance in the allocation totals for the disaggregated waste industries in the Use and Make tables. To prevent this, we apply a different allocation value to the value-added sectors of the disaggregated waste industries based on the intermediate industry totals (i.e., non-value-added allocations):

27 $${\pi }_{W}=(Vi-(i{\prime} (U-W)))/W$$

where *pi*_*W*_ denotes the value-added allocation proportions, *W* is the value added matrix in the form of value added components per dollar industry output that is extracted from the full Use table, *Vi* denotes the industry throughput derived from the Make table (row sums), *i*'(*U*−*W*) denotes the intermediate industry throughput derived from the Use table (column sums) and not including the value added matrix. This adjustment results in different allocation factors from the Use table columns, and are included in the *WasteDisaggregation_Use* sheet of the primary data record in the *VA disaggregation* rows.

#### Disaggregation of the make table

##### Disaggregation of the make table columns

The Make table columns represent which commodities are produced by different industries. There are five industries that produce the 562000 commodity. The *Waste management and remediation services* industry itself produces most of this commodity (83%). Allocation for this sector is described in the Make table intersection disaggregation section. For the rest of the sectors that produce the 562000 commodities, the following assumptions are made.

- *Truck transportation* (484000): the entirety of the 562000 commodity produced by this sector is assigned to the *Solid waste collection* (562111) column, as it is assumed that the truck transportation service is used in waste collection. It is further assumed that the *Hazardous waste collection, treatment, and disposal* sector (562HAZ) collects all hazardous waste using specialized equipment.
- *Services to buildings and dwellings* (561700): the entirety of the 562000 commodity produced by this sector is assigned to the *Remediation services* sector (562910), as it is assumed that the services provided by this sector deal with site-specific remediation.
- *State and local government other services* (GSLO): the 562000 commodity produced by this sector is split between the *Solid waste collection*, *Solid waste landfill*, *Solid waste combustors and incinerators*, and *Material separation/recovery* sectors (562111, 562212, 562213, and 562920). It is assumed that local governments do not perform hazardous waste disposal, site remediation, or other waste disposal functions to a significant degree. The commodity is allocated proportionate to the allocation percentages used for the 562111, 562212, 562213, and 562920 sectors in the Use table row (commodity) totals.

##### Disaggregation of the make table rows

The Make table rows represent which waste management industries (rows) produce commodities (columns) other that waste management. The 562000 commodity represents over 97% of the industry’s total output. For most sectors, the commodity values are distributed using the percentages obtained from the disaggregation of the waste management industries in the Use table. These values are included in the *WasteDisaggregation_Make* sheet in the primary data record, in the *Industry disaggregation* rows.

There are two exceptions to these allocation values in the Make table row disaggregation. For the *Oil and gas extraction* (211000) commodity, the entirety of the production value is assigned to the *Solid waste landfill* (562212) industry, as it is assumed that this represents landfill gas production. For the *Scrap* (S00401) commodity, the entirety of the production value is assigned to the *Material separation/recovery facilities* (562920) industry, under the assumption that these facilities are the ones responsible for recovering scrap during waste management.

##### Disaggregation of the make table intersection

For the disaggregated waste management sectors, the Make table intersection represents the amount of the *Waste management and remediation services* commodities (rows) produced by each of the waste management industries (columns). For the disaggregation procedure, we assume that each disaggregated industry only produces its own disaggregated commodity; in other words, there is no off-diagonal production of waste management services in the intersection. The diagonal-only production assumption is a good first approximation that allows the production impacts from a specific type of waste management service to be assigned to a single sector.

The Make table intersection is allocated using the total Use table commodity output, adjusted for any manual allocations performed for the disaggregation of the Make table columns. These values are included in the *WasteDisaggregation_Make* sheet of the primary data record, in the *Make table intersection* rows.

#### Disaggregation of satellite tables

The satellite tables contain the resource use and environmental releases mapped to the original *Waste Services and Remediation Services* sector. These flows must be adjusted or mapped to reflect the inclusion of the disaggregated waste sectors. To do so, the following approach was taken:

- When the underlying data for specific flows is available at the six digit NAICS level, the flow is mapped to the corresponding disaggregated sector codes as indicated in Table 6.
- When there is additional data available for specific flows which are not adequately reflected at the 6-digit NAICS to USEEIO mapping (as per Table 6), a manual distribution of that data is specified as an input to the disaggregation algorithm. For the *Waste management and remediation services* sector, additional data for the specific flows in the GHG satellite table was assigned using an input file, as specified in the *WasteDisaggregation_Env* sheet of the primary data record.
- When no additional data is available, the satellite flows are disaggregated based on the total economic throughput of the disaggregated sectors. For example, if 562HAZ represents 50% of the total economic value of the disaggregated sectors, then 50% each flow is allocated to 562HAZ.

#### Margin allocations

The margins present for Waste and Remediation were allocated using the total Use table commodity output.

### Sector correspondence

Sector correspondence between the BEA and NAICS codes, or the *Sector Crosswalk*, is created to connect the two classification systems and enable mapping from one system to the other. For v2.0, the Sector Crosswalk is built based on 2012 BEA and NAICS codes and includes 2007 NAICS codes according to the 2012 NAICS to 2007 NAICS concordance by Census Bureau^23^. The Sector Crosswalk is available as part of the primary data record^24^.

The correspondence stems from BEA-NAICS relationship table released with national input-output (IO) accounts by BEA^10^. The relationship table presents a hierarchy of the BEA codes at three levels of detail: sector (21 sector groups), summary (71 sector groups), and detail (405 sector groups), as well as how each level relates to the NAICS code structure. However, the BEA table is insufficient in two aspects:

1. Most BEA codes have explicit correspondence with NAICS codes, but BEA codes in several sector groups, including construction (23), government (G), and final demand (F), are not aligned with specific NAICS industries.
2. Generally, NAICS codes are published by the Census Bureau at five levels of detail: 2- to 6-digit, but such hierarchy is not fully disclosed in the table for each BEA-NAICS correspondence, which makes the correspondence incomplete. For instance, BEA code 1111A0 *oilseed farming* only connects to NAICS 5-digit codes 11111 *soybean farming* and 11112 *oilseed (except soybean) farming* in the table, but in fact 11111 and 11112 have single child codes 111110 *soybean farming* and 111120 *oilseed (except soybean) farming*, respectively, as well as shared parent codes 1111 *oilseed and grain farming*, 111 *crop production*, and 11 *agriculture, forestry, fishing and hunting*. Therefore, BEA code 1111A0 should be connected to all these NAICS codes in order to form a complete BEA-NAICS correspondence.

To solve the first problem, BEA-NAICS correspondence for these sectors is approximated after careful inspection and comparison of their definitions in BEA and NAICS systems. As for the second problem, each BEA-NAICS correspondence is extended to all related NAICS codes based on a Census-released 2–6 digit NAICS Code table^25^. A comprehensive BEA-to-NAICS sector mapping table is built with these complementary pieces to the main correspondence.

While the codes and definitions of commodity categories are not changed from those provided by BEA in v2.0 (except for disaggregated sectors), original names are assigned to the commodity categories to replace the names used in the BEA IO tables. The name changes are made because BEA uses the same names for commodities as those used for the respective industries (industries and commodities share the same codes), which are derived from the NAICS industry names, and the assignment of names better fit to describe a “commodity” rather than an “industry” adds clarity to commodity representation in the model. The new commodity names assigned to the BEA codes are part of the primary data record, in the *commodities_meta* sheet.

### Environmental and employment data modeling

The environmental data inputs for an EEIO model are national totals of flows (physical movements of specific resources, emissions or employment) by industries. Single sources of data for a given flow are generally insufficient for providing environmental and economic performance at the level of resolution required for v2.0’s 400+ industries, and therefore modeling is required to attribute or allocate environmental data from often multiple raw sources to this level of industry resolution. This modeling activity may also be referred to as flow sector attribution modeling. Here we describe updates made for eight satellite tables to reflect new methods for flow sector attribution modeling.

v2.0 builds on the flow sector attribution modeling approach taken to construct national commercial waste totals^8^, by estimating totals by industries defined by NAICS codes. For v2.0, national totals by sector are modeled by NAICS 6-digit codes. This approach is in contrast to the former method used in v1.1 in which flows were directly attributed to IO table industries. The modeling steps were written in Python and consolidated into a software package called *flowsa*. *flowsa* performs extraction, transforming and loading (ETL) processes for bring in original data sources, as well as the modeling steps to create a standard flow-by-sector output, which can be retrieved using the *getFlowBySector* function and passing the name of the flow-by-sector of interest. *flowsa* v1.0.1^26^ was used for preparation of all original environmental inputs.

The environmental datasets not updated since v1.2 include the Commercial non-hazardous waste excluding construction activities, Commercial non-hazardous waste from construction activities, Nitrogen and Phosphorus Releases from Agriculture, Pesticide releases, Mineral extraction, and Energy extraction (Table 2). For these datasets, the national totals by sector were extracted directly from the published datasets^5,7^. All flows in these published datasets, except those from the commercial waste datasets which are waste flows and not elementary flows, were mapped to the FEDEFLv1.0.7. The National GHG Industry Attribution Model^27^ for year 2016 was used for developing the national GHG totals by industry, which is the same flow sector attribution model used for the recently published Supply Chain GHG emission factors^1^, but is an update from the previous GHG satellite tables included in v1 models.

#### Chemical releases to air

Chemical releases to air are sourced from 2017 reported emissions data from the National Emissions Inventory (NEI)^28^ and Toxic Release Inventory (TRI)^29^. Point source releases to air reflect facility reported releases in these datasets and include both criteria and toxic air pollutants. Emissions are assigned to industries based on the NAICS reported by each facility. If a facility reports a NAICS code that is no longer valid, emissions are allocated equally across one or more relevant NAICS from the 2012 schema. Where particular elementary flows are reported in both NEI and TRI, flows are maintained from the NEI only to prevent double counting.

Nonpoint criteria and toxic air emissions are sourced from the 2017 Nonpoint, Nonroad, and Onroad NEI datasets^28^. In these datasets, emissions are reported by county and assigned to source classification codes. In *flowsa*, source classification codes are used to allocation emissions to one or more NAICS through activity-to-sector mapping files. Emissions of pesticides from agricultural activities are excluded from this dataset as they are captured in the pesticides table. The general approach for assigning nonpoint air emissions to sectors remains unchanged since version 1.1. However, revisions to mappings between SCC codes and sectors, and updates in data collection lead to some notable differences in sector emissions including:

1. *Increase in emissions for construction sectors*. A revision to the mapping of NAICS to BEA sectors for NAICS 23 *Construction* fixed an error in v1.1 that resulted in substantially lower emissions coefficients.
2. *Increase in nonpoint emissions for manufacturing sectors*. In past models, nonpoint air emissions from industrial combustion were not mapped to sectors due to insufficient data. In v2.0, these emissions have been allocated to manufacturing sectors on the basis of fuel consumption by fuel type^30^.
3. *Increase in particulate matter emissions for livestock production*. Revised methods for particulate matter estimates in the NEI were implemented since 2011, the data year used in v1.1, that better account for emissions of dust from livestock^31^.
4. *Increase in emissions for landscaping services*. Updates to SCC mapping enable emissions from lawn and garden equipment to be divided between commercial and residential use.
5. *Reduction in emissions for ozone depletion potential for manufacturing sectors*. Emissions from some activities relating to solvent utilization were omitted from this method due to challenges and quality of the allocation approach.

The result is available in the National Criteria and Hazardous Air Pollutant Totals By Industry 2017 v1.1 dataset^32^.

#### Chemical releases to water

Chemical releases to water are sourced from 2017 facility reported emissions data from the Toxic Release Inventory (TRI)^29^ and the Discharge Monitoring Report (DMR)^33^. Chemical releases reported by facilities in these datasets include toxic releases, metal compounds, nutrients, and organic pollutants. Emissions are assigned to industries based on the NAICS reported by each facility to the dataset. Where particular elementary flows are reported in each dataset, flows are maintained from the DMR when a facility reports to both. In v1.1, releases from the DMR were limited to nutrient release of nitrogen and phosphorous. However, in v2.0, releases to water also include organic enrichment, sediments, and other compounds tracked within the DMR. The result is available in the National Point Source Releases to Water By Industry 2017 v1.1 dataset^34^.

#### Chemical releases to ground

Chemical releases to ground are sourced from the Toxic Release Inventory, the same source as used in v1.2. The data have been updated for 2017^29^. Data are assigned to sectors based on facility-reported NAICS. The result is available in the National Point Source Releases to Ground By Industry 2017 v1.1 dataset^35^.

#### Land use

Land occupation is allocated to industrial sectors using the USDA’s Major Uses of Land in the United States 2012 report (MLU), accounting for total US land area through twelve use categories^36^. The MLU land use categories are further allocated to 6-digit NAICS, modeled on Zeng and Ramaswami’s methodology^37^. Estimating industry land use with the MLU as the primary data source is an update from v1.2, where land use was calculated by summing USDA CoA, Bureau of Land Management (BLM) Public Land Statistics (PLS), EIA Commercial (CBECS), and EIA Manufacturing (MECS) land use with the MLU’s statistics for forest land, transportation, national defense, and grazing land^38–42^. In v2.0, these data sources are used to allocate MLU land use categories to relevant sectors. Additionally, there are changes to methods of allocation.

In the original analysis, BLM hard rock leases and EIA MECS relied on BEA employee compensation for granular allocation. In v2.0, these land use categories are allocated to sectors using BLS employment data^43^. Urban land allocation now includes estimates for urban green areas based on assumptions made by Zeng and Ramaswami^37^. EIA manufacturing land area is considered part of the urban land total rather than a stand-alone industrial area category. Land use now differentiates urban and rural residential housing land by incorporating values from the Major Uses of Land report^36^. Land for national parks is directly assigned to NAICS code 712190, where previously, in both the original USEEIO land satellite table and in Zeng’s work, national parks were included in the unaccounted land category. Timberland estimates are based on MLU’s ungrazed forest land rather than total timberland, which reduces land use attributed to forest. The final model is provided in the National Land Occupation Totals By Industry 2012 v1.1 dataset^44^.

#### Water withdrawal

A new water withdrawal sector attribution model was developed, referred to as ‘Water_national_2015_m1’. Water_national_2015_m1 was created primarily using water withdrawal data accessed from the USGS National Water Information System Web Interface^45^. The data includes fresh and saline water withdrawn from surface and ground sources and evaporative water loss to the atmosphere.

The USGS publishes state-level water withdrawal estimates for nine broad categories: Aquaculture, Domestic, Industrial, Irrigation Crop, Irrigation Golf Courses, Livestock, Mining, Public Supply, and Thermoelectric Power. In Water_national_2015_m1, these water withdrawal categories are attributed to sectors using additional data sources for allocation, when necessary.

Five water withdrawal categories are directly attributed to sectors. Aquaculture and Thermoelectric withdrawals are assigned to the 4-digit NAICS codes 1125 and 2211, respectively. Water flows are equally allocated to all related 6-digit NAICS. Domestic water withdrawals are assigned to the BEA code F01000, as no NAICS code represents households. Irrigation Golf Courses water withdrawals are assigned to NAICS 713910. Net public supply is calculated by subtracting public supply deliveries to domestic to avoid double counting, then assigned to NAICS 221310.

The attribution methodology for the remaining water categories follows Rehkamp *et al*.’s sector attribution approach^46^. Industrial and Mining water withdrawals are proportionally allocated using BLS QCEW employment data^47^. Crop Irrigation water withdrawals are allocated proportionally using water use by crop type. Crop water use is calculated by multiplying irrigated harvested cropland acreage and water application rates for different crops^48,49^. For states that do not distinguish between irrigation used for crops and golf courses, all irrigation water is attributed to crops. Livestock water withdrawals are allocated proportionally using water use by animal type, calculated from the USDA animal inventory and national median water intake rates by animal type^48,50^. The water withdrawal sector attribution model result is published as the National Water Withdrawal Totals By Industry 2015 v1.1 dataset^51^.

The Water_national_2015_m1 model differs from the Water Use Satellite table compiled for v1.1^3^ in several ways. The water methodology in the Water Use Satellite table compiled for v1.1 tracked water returns, allowing for the calculation of water consumption by industry. The Water_national_2015_m1 model does not include water returns, as available estimates for water returns are from 1995 and do not account for advances and updates in machinery^52^. Additionally, the Water_national_2015_m1 methodology differs from Water Use Satellite table compiled for v1 for Crop Irrigation, Industrial, Mining, Thermoelectric, and Hydroelectric water estimates. Water use for crops originally used acreage data for 37 crops accessed through the USDA Irrigation and Water Management Survey (IWMS)^49^. To achieve more granular results for 6-digit NAICS, Water_national_2015_m1 uses acreage data for 64 crops from the USDA Census of Agriculture (CoA) data^48^. Both versions of sector attribution modeling use the IWMS statistics on water application rates. Industrial water withdrawals in v1.1 were calculated by scaling Canadian water withdrawals for manufacturing by US GDP. In Water_national_2015_m1, employment data are used to allocate water withdrawals to relevant sectors identified by USGS. The original Mining attribution version calculated results using process and employment-based factors published by Blackhurst *et al*.^53^. As natural gas withdrawals have increased 37% between 2002 and 2015 in the United States, the 2002 values likely are not representative of current water requirements for the mining sector^54^. In Water_national_2015_m1, mining water withdrawals are allocated to 6-digit NAICS using employment data published by BLS QCEW. Water_national_2015_m1 no longer includes water withdrawal for hydroelectric power, as the USGS stopped estimating national water withdrawals for this category in 2000^55^.

#### Commercial RCRA-defined hazardous waste

Data for commercial hazardous waste are sourced from the Resource Conversation Recovery Act Biennial Report, the same source as used in v1.2. They have been updated for 2017^56^. Data are assigned to sectors based on facility-reported NAICS. The result is available in the National Commercial Hazardous Waste Totals by Industry 2017 v1.1 dataset^57^.

#### Employment

The employment sector attribution model is created by importing and formatting the 2017 BLS Quarterly Census of Employment and Wages (QCEW) table^58^. QCEW publishes national annual employment at the 6-digit NAICS; no additional allocation is required for use in v2.0. Where the BLS is missing data, less aggregated NAICS are summed, or more aggregated NAICS are equally allocated. The model is available as the National Employment Totals By Industry 2017 v1.1 dataset^59^. The earlier versions of USEEIO used the BLS National Employment Matrix, which also publishes employment data by NAICS sector codes^5^. QCEW was chosen for the sector attribution model, as QCEW data is one of the primary data sources for the National Employment Matrix and as the National Employment Matrix database primary purpose is for national-level employment predictions^60^. Additionally, QCEW publishes state and county employment data used in other sector attribution models used in USEEIO v2.0. Using BLS QCEW for the employment model allows for a consistent data source for all employment data used throughout model construction.

#### Value added

Value added is a collection of the monetary benefits industries provide to government (as taxes), employees (as wages), and to their shareholders (as profits). For v2.0, total value added per industry is taken directly from the same 2012 BEA Use table that is a source for the economic data. Although it is economic data, the handling of it is identical to that of the environmental and employment data used to construct the satellite tables. This is an update from v1.1, where value added data were taken from BEA Summary level Use tables for more recent years and adjusted as described in the documentation^5^.

### Procedure for model building

The R package *useeior* v1.0.0^61^ was used for USEEIO v2.0 model creation. A model configuration file was first created to define all the model input data and characteristics. The waste sector disaggregation procedure required the definition of an additional set of configuration files that provide instructions for this disaggregation procedure. Once all the requirements are installed, the generation of v2.0 takes place in a single *buildModel* function to load the various data components and build the model. The original environmental and employment data were all produced by from *flowsa* v1.0.1^26^, whereas environmental datasets originally created for v1 USEEIO models^5,7^ were mapped and reformatted. All the indicator datasets were produced by the *LCIA formatter* v1.0.1^62,63^, which like the environmental and employment data had been harmonized with the FEDEFL. All these data products were uploaded to the EPA Data Commons^64^ and are retrieved by *useeior* during model building as specified in the model configuration file. All the economic input data (Table 1) are retrieved using scripts in *useeior* and saved in the package along with selected indicators not from *LCIA formatter* (jobs, value added, and waste indicators, Table 3), commodity names, data inputs to the Sector Crosswalk, and model metadata files. Model validation and output writing are also performed in simple statements. The commands for building v2.0 with *useeior* v1.0.0 along with the results of the validation procedure are shown in a model building and validation document^65^.

## Data Records

Table 7 lists the original data records produced for v2.0, along with their associated use in the model. The USEEIO v2.0.1–411 dataset is the primary data record, and includes the waste disaggregation data inputs, model components, result matrices, price adjustment matrices, and demand vectors, along with supporting metadata including sector, flow and indicator descriptions. The additional data records are national flow totals by sectors that serve as data inputs in model building.

**Table 7:** Original data records created for USEEIO v2.0.

Dataset	Note
USEEIO v2.0.1–411^24^	Complete Model
National Water Withdrawal Totals By Industry 2015 v1.1^51^	Environmental Input
National Criteria and Hazardous Air Pollutant Totals By Industry 2017 v1.1^32^	Environmental Input
National Point Source Releases to Ground By Industry 2017 v1.1^35^	Environmental Input
National Point Source Releases to Water By Industry 2017 v1.1^34^	Environmental Input
National Commercial Hazardous Waste Totals by Industry 2017 v1.1^57^	Environmental Input
National Land Occupation Totals By Industry 2012 v1.1^44^	Environmental Input
National Employment Totals By Industry 2017 v1.1^59^	Environmental Input

## Technical Validation

### Economic validation

The disaggregation of the Waste Sector introduced changes to the economic transactions present in the 2012 BEA Detail Make and Use Tables that is used as the basis of the USEEIO model. To ensure that these changes do not violate the balance required to build the model, both the commodity and industry totals of the Make and Use tables were compared after the disaggregation process to the original datasets in a commodity-to-commodity and industry-to-industry comparison. The v2 industry output and commodity output totals for each commodity and industry in the model were both found to be within 1% of the original totals. In the case of the new waste commodity and industry totals, they summed to within 1% of the *Waste and Remediation* commodity and industry totals in the 2012 BEA Detail Make and Use tables.

### Environmental and employment flow validation

Environmental flows generated in *flowsa* are checked for data loss after allocation to industry sectors, by comparing flow amounts in the original source data to flow amounts in the final output. Environmental flows are transformed from source data schema, typically NAICS 2012 codes, to USEEIO schema (e.g. BEA detail) through a schema mapping. Following this mapping, each satellite table is assessed to ensure that no environmental flows were lost during the transformation. Additionally, flow amounts for each flow within a satellite table must not change more than 0.05%. Flow loss may result from invalid NAICS codes. Following transformation of all satellite tables, environmental flows are compared across satellite tables to check for potential duplication. This ensures that, for example, pesticide releases to air are not duplicated in both the Criteria and Hazardous air pollutant satellite table and the pesticide satellite table. In some cases, environmental flows may appear in more than one satellite table if the associated sectors do not overlap. For example, releases of nitrogen and phosphorous are sourced from the Nitrogen and Phosphorus Release from Agriculture satellite table (NPAG) specifically for agricultural sectors, while data for all other sectors are sourced from the Discharge Monitoring Report via the Point source releases to water satellite table (WATREL).

### Model validation

Checking that national flow totals by sector used as inputs to the model can be recalculated using appropriate model components serves as the primary means of full model validation. This check is performed by using Eq. 28.

28 $${E}_{c}={B}_{\chi ,c}L\widehat{y}$$

The left side is the original flow by industry totals, *E*_*i*_, put into a flow x commodity form, *E*_*c*_. *E*_*i*_, a national total of flow by industry per year consisting of the concatenation of all the satellite tables described above, is available in varying years. *E*_*c*_ is obtained from *E*_*i*_ by multiplying its transpose by the commodity mix matrix, *C*_*m*_, and transposing the result.

29 $${E}_{c}={({C}_{m}{E}_{i}^{{\prime} })}^{{\prime} }$$

30 $${C}_{m}={V}^{{\prime} }{\widehat{x}}^{-1}$$

*C*_*m*_ is obtained from Eq. 30, where *V'* is the transposed model Make table, which is normalized by multiplying it by the diagonalized form of the inverse of model output, *x*.

The right side of Eq. 28 is a slightly modified form of the model result calculation using the direct perspective. *B*_*i*_ is the satellite matrix in industry form from Eq. 3.

31 $${B}_{\chi ,c}={B}_{i}\,\circ \,\chi V{\widehat{q}}^{-1}$$

As the original flow totals in *E*_*i*_ are in various dollar years but the model economic components are all in a consistent 2012, to validate the model, an output adjustment is required to *B*_*i*_, which is achieved through multiplication with *χ*, an output adjustment matrix, as well as transforming it to commodity form. This equation is shown in Eq. 31. *χ* is composed of *x*_*s*_*:x* output ratios and identical in its indices (rows and column identifiers) to *B*. The element-wise product of *B* and *χ* adjusts B for the flow year differences and effectively converts *B* into a harmonized 2012 year form. This then must be further transformed into commodity form before use, which is done so by multiplication with the market shares matrix, *V*_*n*_, which itself is obtained from the model Make table and the model commodity output, *q*.

The validation results show that the model passes the check shown in Eq. 28 for all flows at a 1% tolerance, with a <1% difference assumed to be expected due to rounding errors^65^ both for the full model and the domestic model, where *L*_*d*_ Eq. 25 is substituted for *L* and *y*_*d*_ Eq. 23 is used for *y*. The economic outputs also checks for both models, except for the commodities *Used and secondhand goods* and *Noncomparable imports*, which have negative uses for balancing purposes in the Use table and therefore failures were expected. Results for these commodities should not be used for analytical purposes.

A summary of life cycle impact assessment results from v2.0 for 2012 total US production and consumption are presented in Table 8. The calculated per capita GHG in the US production view of 15.57 MTCO2e/person is reasonably close to a World Bank tabulated estimate of 15.77 MTCO2e/person in 2012^66^, considering USEEIO v2.0 is a mixed year model with the GHG emissions data representing 2016 intensity in 2012 USD.

**Table 8:** USEEIO v2.0 Life cycle impact assessment results of total US Production and Consumption.

Indicator	Unit	US Production 2012 - Total	US Consumption 2012 - Total	US Production 2012 - Per Capita	US Consumption 2012 - Per Capita
ACID	kg SO2 eq	1.17E + 10	1.20E + 10	37	38
CCDD	kg	4.84E + 11	4.85E + 11	1533	1533
CMSW	kg	1.91E + 11	1.93E + 11	606	611
CRHW	kg	3.37E + 10	3.59E + 10	107	114
ENRG	MJ	8.19E + 13	1.20E + 14	259148	378921
EUTR	kg N eq	6.83E + 09	6.61E + 09	22	21
ETOX	CTUe	3.94E + 12	3.75E + 12	12477	11869
WATR	kg	2.82E + 14	2.98E + 14	893859	942503
GHG	kg CO2 eq	4.92E + 12	5.17E + 12	15568	16374
HRSP	kg PM2.5 eq	2.60E + 09	2.51E + 09	8	8
HTOX	CTUh	5.07E + 04	5.44E + 04	0	0
LAND	m2*yr	8.22E + 12	8.87E + 12	26012	28077
MNRL	kg	2.24E + 12	2.28E + 12	7101	7210
OZON	kg CFC-11 eq	8.64E + 05	9.59E + 05	0	0
SMOG	kg O3 eq	1.12E + 11	1.23E + 11	354	388

Per capital results assume a US population of 3.16E8 persons. The full names of the indicators are given in Table 3. Full results can be found online^73^. Zero values appear due to rounding, actual values are between 0–0.5.

#### Comparison of USEEIO v2.0 with USEEIO v1.2

As v1.2 represents the most recently previously peer-reviewed and published USEEIO model, but was built with a different set of data inputs and a different software procedure, comparing v2.0 results against v1.2 is a relevant means of performing model validation. It may also yield insights into changes in US industry environmental performance that may be of interest to users that have used a v1 model either directly or via an interface like the SMM Prioritization Tools. The v1.2 model data used here for comparison were acquired from the USEEIO-API. A selected comparable model result matrix (N) and sector rankings derived from full model LCIA calculations are appropriate for this validation and comparison, because they represent measures resulting from the combination of all model components both at the unit scale (impact per USD) and as a result of total US production and consumption.

##### Direct + Indirect Impact Intensity Comparison

Shifts in sector impact intensity (as present in the *D* and *N* matrices) between v1.2 and v2.0 are attributed to a combination of changes in methodology for resource allocation to industry sectors, primary and allocation data sources, source data years, and economic growth or decline within industries between the related source data years, after the currency year adjustment Eqs. 4, 5. We examine these differences by indicator in a series of grouped charts comparing v2.0 and v1.2 impact coefficients (*N* matrix) by sector and indicator^67^. An analogous series of direct impact coefficient (*D* matrix) comparison charts are also provided for interpretation^68^, but are not analyzed in depth here. The reader should refer to Table 3 for the source of the impact method characterization factors used to construct the *N* and *D* matrices.

Collectively there are small decreases in ACID in most sectors. The decrease is more notable in electricity and transportation sectors. ACID in utilities, manufacturing and transportation sectors is largely driven by criteria air pollutant emissions like sulfur dioxide (SO_2_) and nitrogen oxides (NO_X_). For *Electricity*, for example, SO_2_ and NO_X_ contribute to 57% and 39% of impact, respectively. This general decrease in v2.0 factors reflects the steady national decrease in SO_2_ emissions from 2011 to 2017^69^.

Changes in GHG intensity were less than 0.5 kg CO_2_e/$ for >95% of sectors. The largest visible change in GHG intensity was seen in the electricity sector, with a nearly 2 kg CO_2_e/$ decrease. This decrease is likely a result of fuel source changes in the electricity production over this period^69^. Natural gas also saw a larger decrease relative to other sectors.

Parallel decreases to that seen in ACID can be seen in the SMOG indicator. SMOG impacts are driven by emissions of NO_2_ and volatile organic compounds (VOCs). For SMOG, decreases are also apparent in agricultural sectors and many manufacturing sectors where decreases in impact intensity for *Wood pulp*, *Paints…* and *Cement* stand out. In agricultural sectors, the consumption of other agricultural commodities are the primary drivers of SMOG. Direct SMOG impacts have decreased across most agricultural commodities, especially crops (e.g. *Fresh soybeans, canola, flaxseeds, and other oilseeds* and *Fresh wheat, corn, rice, and other grains*). Additionally, the use of *Electricity* and *Truck transport* by agricultural sectors are the greatest contributors to SMOG from non-agricultural sectors. As described above, direct SMOG impacts have decreased substantially in these two sectors specifically.

HRSP impacts are driven by particulate emissions, and to a lesser extent in agricultural sectors, ammonia emissions. Significant decreases in HRSP are visible in agricultural sectors except in *Cattle…*. Direct HRSP impacts in agricultural sectors for crops account for a significant share of total impacts (e.g. 95% for *Fresh soybeans, canola, flaxseeds, and other oilseeds* and *Fresh wheat, corn, rice, and other grains*). In v2.0, direct impacts decreased between 30–50% for crops. The increase in HRSP for *Cattle…* in v2.0 is due to the improved modeling of dust from livestock^31^. This significant decreases in agricultural sectors is carried over into the food and fiber sector products, where for instance 90% of the contribution to HRSP for *Flours and Malts* is from *Fresh wheat, corn, rice, and other grains*.

OZON showed decreased or little change in nearly all sectors. The Criteria Air and GHG emissions data from 2017 and 2016, respectively, that drive these data replaced 2011 and 2013 data in v1.2 and likely reflect the continued phase out and substitution of ozone depleting substances^70^. However, this interpretation does not hold for the pesticides that contribute to this impact, which include Methyl Bromide. In the v2.0, *methyl bromide/emission/air/troposphere/rural/ground-level/kg* has one of the highest CFC-11 equivalents (0.51, sheet *C*, cell *BMV20* of)^71^ of all flows, and *Fresh vegetables, melons, and potatoes* shows the highest at 2.4E-5 kg/$. The equivalent value is 1.44E-5 in v1.2 (sheet *B*, cell *D681* of)^71^. A similar effect can be seen in the increase in ETOX for *Fresh vegetables, melons, and potatoes* which is also driven by pesticides, where.lambda.-Cyhalothrin, Chlorothalonil, and Cyfluthrin contribute 27%, 22%, and 19%, respectively, to this impact. The national release estimates for pesticides were not updated in v2.0, where data on application for these chemicals to vegetables was from 2010. With the pesticide loss model input data remaining the same, but inflation in the commodity as seen in the *P* matrix between 2012 (USD year of v2.0) and 2013 (USD year of v1.2) created a lower denominator in v2.0, resulting in a higher pesticide-related impact intensity (since dollar output is in the denominator) for this sector. Other agricultural commodities show the inverse change in v2.0, where the agricultural output in v2.0 is higher and thus the pesticide release and related impact intensities are lower.

Some changes in HCAN and NCAN result from the inclusion of characterization factors from TRACI 2.1 for metals, which were not included for v1.2. Mercury emissions to air drive the increase seen in *Cement* (9% contribution to HTOX), that are not due to change in emissions, but rather toxicity characterization that was not present in v1.2.

The mineral extraction data driving the MRNL impacts was not updated in v2.0. For MRNL, the only notable changes in the use intensity are the decrease in *Dimensional stone* and increase in *Sand, gravel, clay…* This can be explained by an error in allocation of the *Sand/gravel* flows to *Dimensional stone* rather than to *Sand, gravel, clay… in v1.2 and prior versions.*

Water withdrawal impact intensity differences are attributed to allocation methodology changes for the irrigation, mining, and industrial USGS water use categories. Crop irrigation water withdrawal is initially calculated by determining water withdrawal for individual crops. In v1.2, withdrawals were calculated for 37 crops published in the 2008 USDA Irrigation and Water Management Survey (IWMS). In v2.0, withdrawals are calculated for 64 crops identified in the 2017 USDA CoA. The change in crop categorization coupled with differences in survey responses resulted in changes in industry impact intensity. The decline in impact intensity for *Tobacco, cotton, sugarcane, peanuts, sugar beets, herbs and spices and other crops* is attributed to correcting an error in the v1.2 calculation. v1.2 estimated 2007 mining water withdrawals by scaling 2002 water IO vector coefficients in gal/$M by changes in annual employment^53,71^. Due to shifts in the oil and gas industry towards increased natural gas extraction, the 2002 water withdrawal coefficients likely do not reflect 2015 water withdrawals for mining industries. In v2.0, water for mining sectors is first attributed to 6-digit NAICS using employment data before mapped to BEA industry codes. The allocation methodology for industrial water withdrawal was modified for v2.0. In the original analysis, industrial water was allocated to NAICS 31–33 using 3-digit NAICS Canadian Industrial Water Use statistics, scaled to US production by US GDP. In v2.0, the industrial water withdrawal sectors were expanded to include sectors within NAICS 11, 23, 48, 51, 54, 56, and 81, as defined in the USGS to NAICS crosswalk in *flowsav1.0.1* and as recommended by the USGS. Water withdrawal by industry was allocated to NAICS using BLS QCEW employment data. Expanding the definition of industrial water use allowed for calculation of impact intensities for industries not previously captured, such as industries within wholesale trade, retail trade, and professional and business services.

The greatest discrepancies in land use impact intensities between the two models occur in agricultural industries, with a decrease in intensity for *Cattle ranches and feedlots* and an increase in intensity for *Animal farms and aquaculture ponds (except cattle and poultry)* and *Timber and raw forest products*. Outside of agricultural land, there is a sharp increase for *Museums, historical sites, zoos and parks*. These variances are due to methodology changes when allocating land use to 6-digit NAICS. Changes in animal-related land impact intensity are due to modifying the allocation method for grazing land. In v1.2, national land use by animal type were calculated by importing and summing state level data for “land in farms” from USDA CoA. The difference between the summed state level data and published national MLU pasture and grazed land data were attributed to animal type using a national average of the USDA CoA data. The USDA CoA state data was summed with the allocated MLU remainder land for total grazing land by animal. In v2.0, the USDA CoA “land in farms” data are used as an allocation source, rather than as a primary data source. State level USDA CoA data are used to calculate fractions of land use by animal type, which are multiplied by state level MLU pasture and grazed land. The state data are summed to calculate national land use by animal type for pasture and grazed land. *Timber and raw forest products* impact intensity increased due to a decrease in land area assigned to timberland and negative economic growth between 2007 and 2012. In v1.2, MLU’s “Total Timberland” was assigned to timberland, while in v2.0, MLU’s “Forest-use land not grazed” was assigned to timberland. This methodology modification follows work by Zeng and Ramaswami and is meant to avoid double counting forest land use with grazing land^37^. The increase in impact intensity for *Museums, historical sites, zoos and parks* is explained by accounting for national parks in land use v2.0, whereas previously land for national parks was excluded.

There are few notable changes in intensity of CRHW in v2.0. *Corn products* shows a substantial increase in total CRHW relative to similar sectors. Direct releases from this industry remain fairly consistent from the prior model. However, changes in the Make Transactions have resulted in a substantial increase in the quantity assigned to the *Corn products* commodity; *Other basic organic chemicals*, which is one of the largest generators of CRHW, supplies a significantly higher share of *Corn products* in the make transactions, increasing from approximately 4% of total commodity output in 2007 to 37% in 2012.

The variation between v1.2 and v2.0 employment impact intensities are due to changes in data year and source. v1.2 was created using the 2014 BLS National Employment Matrix, while v2.0 was created with 2017 BLS QCEW. Although QCEW employment data is one of the main sources in the creation of the National Employment Matrix, the National Employment Matrix also incorporates data from the Occupational Employment Statistics program (OES), the Current Employment Statistics program (CES), and the Current Population Survey (CPS)^60^. The modified methodology results in significant sector disparities within agriculture, construction, retailing, finance, and household sectors. For example, the 2014 QCEW estimates 279 thousand private household employees, while the National Employment Matrix estimates 821 thousand employees. v2.0 relies on the BLS QCEW data for the employment satellite table to maintain a consistent employment data source throughout all environmental accounts, as BLS QCEW is used as an allocation source.

For v2.0, value added direct and indirect impact coefficients from *N* are ~1 for all sectors. This follows from the use of the value added directly from the 2012 Use tables and the Leontief price model^72^ that the sum of all direct and indirect value added equals the index price. If the index price is assumed to be equal to the sum of the inputs, then that index price is ~1. This can be represented in unit form in Eq. 32, where the sum of industry value added, *w*, after normalization and transformation to be in commodity form, multiplied by the total requirements matrix, *L*, results in ~1 for each sector, *i*.

32 $$i=w{\widehat{x}}^{-1}V{\widehat{q}}^{-1}L$$

33 $$w=iW$$

The values in v2.0 resulting from Eq. 32 are the same as the “Value Added” row values in the model *N* matrix. ~98% of commodities have a value of 1 ± 0.025. In contrast, in USEEIO v1.2, the comparable value added coefficients varied from ~0.5–1.5, because of a method to approximate 2016 value added from summary level data to pair with the 2007 Use table^6^ that led to a departure from this identity.

##### Ranking comparison

Ranking sectors based a composite score of selected total impacts associated with total US demand is used as a means of prioritization in the SMM Prioritization Tools. Comparing rankings may also be used as another form of model validation that incorporates the demand vectors and the indicators as well as the model result matrices. The composite score for the rankings are calculated as a sum of fractions of sector impact relative to total impact across all sectors by each selected indicator, and then this fraction for a sector was summed across all indicators. This can be represented using Eq. 34, where *z* is a vector of scores for each commodity, and *h*, calculated in Eq. 35, is a vector of the column sums of the given *H* (see Eqs. 17, 19) matrix, representing total indicator amount for each indicator.

34 $$z=(H{\widehat{h}}^{-1})i{\prime} $$

35 $$h=iH$$

**Fig. 1:**
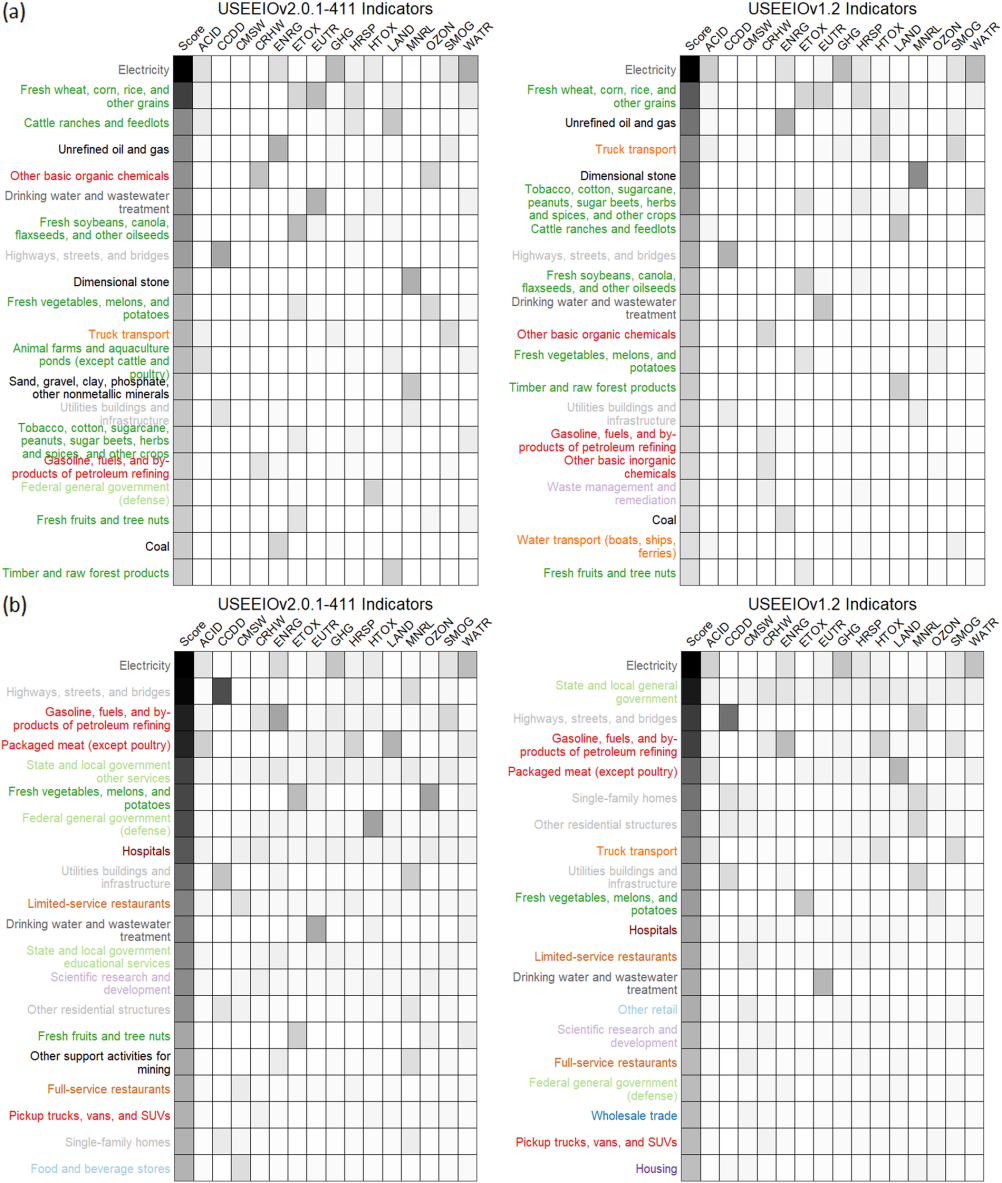
Top 20 commodities by composite impact score for models v2.0 and v1.2 calculated using (**a**) the total US production demand vector and the direct perspective and (**b**) using the total US consumption demand vector and the final perspective. Darker shade indicates a relatively higher score. The full names of the indicators in the columns are given in Table 3.

The first ranking uses *H*_*r*_ calculated where *y* is the US production vector, *y*_*p*_ (see Eq. 14). The second ranking is done with *H*_*f*_ calculated where *y* is the US consumption vector, *y*_*c*_ (see Eq. 13). The same default set of indicators used in the SMM Prioritization Tools were used in this ranking for both models. Results are presented in Fig. 1. Complete *H*_*r*_ and *H*_*f*_ matrices with results for all indicators and by sector are available online^73^.

The sets of commodities in the top 20 from v2.0 and v1.2 in the production and consumption-based rankings are nearly identical, with some notable substitutions and some exchanging of places. In Fig. 1a, E*lectricity* followed by *Fresh wheat, corn, rice, and other grains* remain in the top two places, but *Cattle ranches and feedlots* has moved into the third place. *Other basic organic chemicals* leapt 6 places, while *Truck transport* fell 7 places. *Waste management and remediation services* fell out of the top 20 due to the disaggregation of the waste sectors in v2.0. *Tobacco, cotton, sugarcane, peanuts, sugar beets, herbs and spices, and other crops* fell 14 places, apparently to decreased water consumption.

In Fig. 1b, rankings reveal some minor shifting of positions. *State and local general government* is split into education and other services in the 2012 IO tables, resulting in a fall in ranking but occupying two spots in the top 20. *Federal government (defense)* climbed from 17th to 7th due to an increased relative amount of HTOX. *Fresh vegetables, melons, and potatoes* climbed from 10th to 6th position. *Hospitals* and *Limited Service Restaurants* have moved up in the rankings. *Truck transport* fell out of the top 20. *Food and beverage stores* appear in v2.0 rankings as the only retail sector, whereas *Other retail* appeared as the only retail sector in the v1.2 top 20. The construction sectors, *Single family homes*, *Other residential structures*, and *Housing*, have all dropped significantly in the ranking, the latter two no longer appearing in the top 20.

The overall consistency in the impact intensities and rankings between v2.0 and v1.2 confirms relative consistency and robustness in the model with some changes that can be explained based on input data changes or methodological improvements.

##### Domestic proportion of impact

As described in the Splitting Impacts… section, in v2.0, impacts can be split between those originating in the US vs. the rest of the world.

**Fig. 2:**
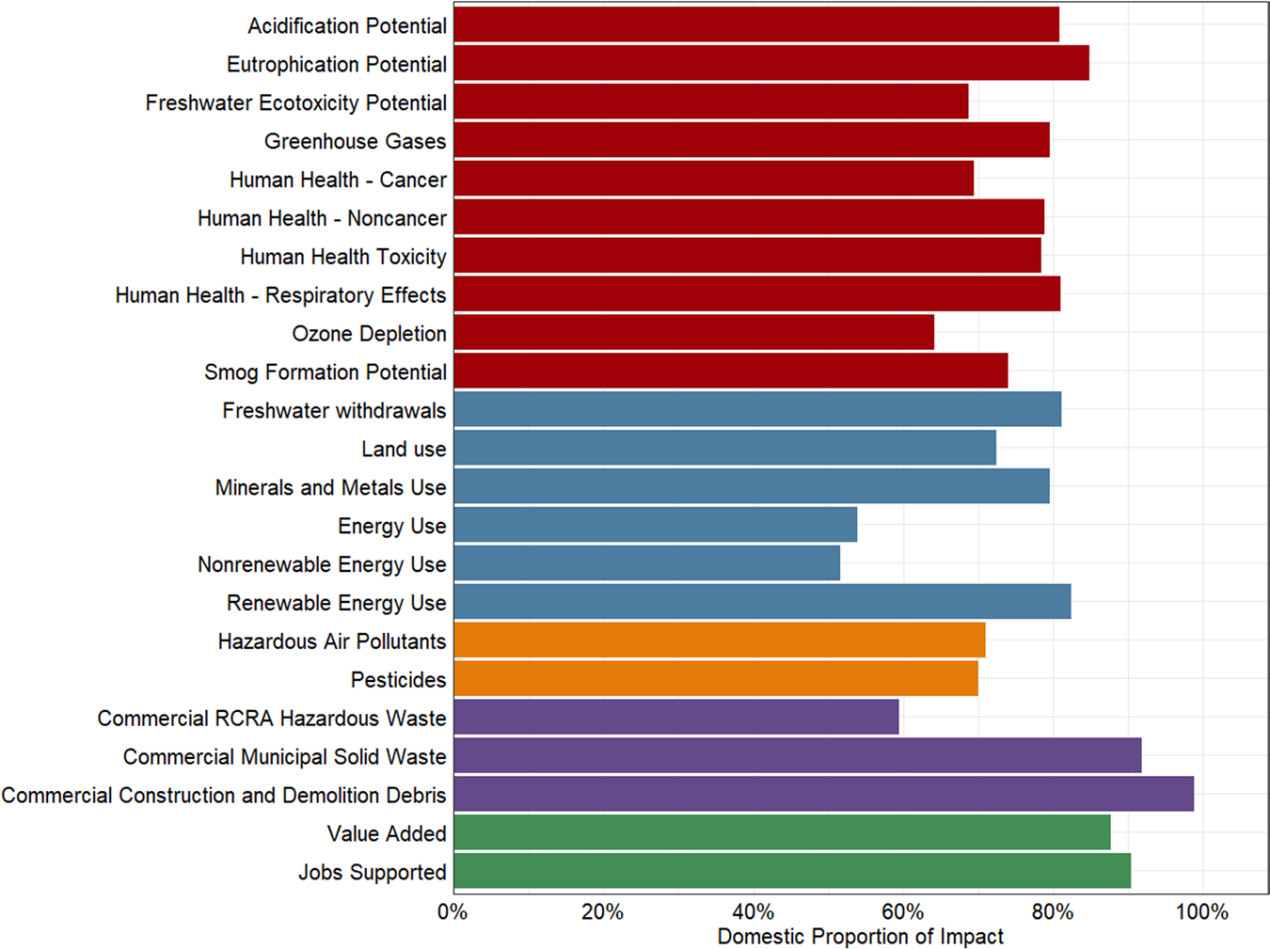
Domestic Proportion of the Impacts of US Consumption.

The accuracy of the impact proportion depending on the validity of the assumption that domestic impact intensities are equivalent to foreign impact intensities, which is not likely valid in all cases. However, Fig. 2 shows that domestic impacts are indeed a proportion of total impacts, with proportions varying by indicator, due to the degree of requirements being met by processes driving the respective impacts. Pairing the domestic proportion with the ranking figure (Fig. 1b) that draws on the same calculation result, we can see that impacts with high domestic proportions to impact, like *Greenhouse Gases*, *Eutrophication*, *Commercial Construction and Demolition Debris* are dominated by sectors such as utilities (e.g. *Electricity* and *Drinking Water*) or construction activities (e.g., *Highways, Streets and Bridges*, and *Utilities Buildings and Infrastructure*), which are sectors dominated by domestic activities. Impacts with smaller domestic proportions of impact like *Energy Use* are dominated by sectors like *Gasoline, fuels…*, that are more dependent on imports for their inputs, as in crude oil for this sector in 2012^74^.

## Usage Notes

Prioritization of sectors in the US economy by greatest opportunities for environmental improvements through the use of the Sustainable Materials Management Prioritization Tools will be a primary use of v2.0. This use applies the same ranking approach used in Fig. 1.

The columns of the *A* or *L* matrices show the pure economic direct or total requirements for a commodity, which may be used for supply chain mapping or for economic impact analysis. Any of these matrices, used together with its respective domestic matrix, can be used to divide the values between those occurring in the US and those in the rest of the world, by taking the difference of a result with the domestic model and the full model, as in Eq. 26. Users should be aware of the limitations of using the Rest of World results. While the model does cover these impacts, they are modeled with a domestic technology assumption, which assumes imports are produced with the same inputs and produce the same emissions per dollar commodity as US commodites.

The footprint of US consumption or production, measured in GHGs, water, or any of the 20 + indicators present in Table 3 can be calculated using the model. These footprints can be calculated by performing a model calculation as in Eqs. 15, 18

The *M* or *N* coefficient matrices may be used for estimating direct plus indirect (supply chain) impacts or embodied carbon, energy, land or water associated with purchases. The *B* or *D* matrices may be used for similar purposes but only include the direct impact or flow per USD. Users can find a coefficient (per USD) in producer’s price in 2012 USD by finding the cell at the intersection of the row with the flow (*M* matrix) or indicator (*N* matrix) of interest along with the column with the commodity best representing the purchase. The adjustment matrices (*P* and *Φ*) can be used as desired to transform a coefficient into a more recent dollar year and into purchaser price. These coefficients can then by multiplied by the cost of the purchase of interest to get the respective total flow or impact associated with that cost in purchaser’s price. The calculation of total impact, *H*, measured with indicator, *i*, associated with a purchase of commodity, *c*, in year *y* USD, with the cost of the commodity *$*_*c*_ given in purchaser price would be calculated with Eq. 36.

36 $${H}_{i,c}={{\rm{\$}}}_{c}{N}_{i,c}{P}_{c,y}{\varPhi }_{c,y}$$

In order to perform this calculation, the purchase has to be associated with a corresponding commodity in USEEIO. The Sector Crosswalk can be used to identify a NAICS code associated with a USEEIO code, and tools like the Census NAICS code search^75^ can be used to identify NAICS codes associated with the purchase.

USEEIO models are under continuous revision with intermittent releases. Real time updates can be found in the *useeior* software repository. Reviewed and released models are listed on the model technical content webpage .

**Table 9:** Flow-by-sector methods from *flowsav1.0.1* used to create the corresponding national flow totals by sector models.

Dataset	flow-by-sector method
National Water Withdrawal Totals By Industry 2015 v1.1	Water_national_2015_m1
National Criteria and Hazardous Air Pollutant Totals By Industry 2017 v1.1	CAP_HAP_national_2017
National Point Source Releases to Ground By Industry 2017 v1.1	GRDREL_national_2017
National Point Source Releases to Water By Industry 2017 v1.1	TRI_DMR_national_2017
National Commercial Hazardous Waste Totals by Industry 2017 v1.1	CRHW_national_2017
National Land Occupation Totals By Industry 2012 v1.1	Land_national_2012
National Employment Totals By Industry 2017 v1.1	Employment_national_2017

### Disclaimer

The U.S. Environmental Protection Agency, through its Office of Research and Development, funded and conducted the research described herein under an approved Quality Assurance Project Plan (K-LRTD-0030017-QP-1-3). It has been subjected to the Agency’s peer and administrative review and has been approved for publication as an EPA document. Mention of trade names or commercial products does not constitute endorsement or recommendation for use.

**List of Acronyms**

ACID - Acidification Potential

BEA – Bureau of Economic Analysis

BLM - Bureau of Land Management

BLS - Bureau of Labor Statistics

CalRecycle - California’s Department of Resources Recycling and Recovery

CBECS - Commercial Building Energy Consumption Survey

CCDD – Commercial Construction & Demolition Debris

CMSW - Commercial Municipal Solid Waste

CoA - Census of Agriculture

CRHW - Commercial Resources Conservation and Recovery Act-Defined Hazardous Waste

DMR - Discharge Monitoring Report

EEIO - Environmentally-Extended Input-Output

EIA – Energy Information Administration

ENRG - Energy Use totals

EUTR - Eutrophication Potential

ETOX - Freshwater Ecotoxicity Potential

GHG - Greenhouse Gases

HAPS - Hazardous Air Pollutants

HCAN - Human Health - Cancer

HNCN - Human Health - Noncancer

HTOX - Human Health Toxicity

HRSP - Human Health - Respiratory Effects

JOBS - Jobs Supported

IO - Input-Output

IWMS - Irrigation and Water Management Survey

LAND - Land use

LCA – Life Cycle Assessment

LCIA - Life Cycle Impact Assessment

LCI - Life Cycle Inventory

MECS - Manufacturing Energy Consumption Survey

MNRL - Minerals and Metals Use

MLU - Major Uses of Land

NAICS - North American Industry Classification System

NEI - National Emissions Inventory

OZON - Ozone Depletion

PEST - Pesticide loss totals

PLS - Public Land Statistics

RCRA – Resource Conservation and Recovery Act

RCRAInfo - Resource Conservation and Recovery Act Information system

RoW - Rest of World

SCC - Source Classification Codes

SMM - Sustainable Materials Management

SMOG - Smog Formation Potential

TRI - Toxic Release Inventory

USD – US Dollars

USDA – US Department of Agriculture

USEEIO – United States Environmentally-Extended Input-Output Model

USEPA – United States Environmental Protection Agency

USGS – US Geological Survey

VADD - Value Added

WATR - Freshwater withdrawals

## Data Availability

USEEIO v2.0 was built in *useeior v1.0.0*^61^. The environmental and employment datasets were created with *flowsa v1.0.1*^26^ as flow-by-sector data products. The flow-by-sector method names for the corresponding datasets are shown in Table 9. The indicator characterization factors for all elementary flows were built in the *LCIA Formatter v1.0.2*^63^ as LCIA data products. The USEEIO Modeling Framework for USEEIO v2.0^9^ provides an overview of the source code along with links to *useeior* and supporting software packages.
